# Shifting trends and age distribution of ESKAPEEc resistance in bloodstream infection, Southwest China, 2012–2017

**DOI:** 10.1186/s13756-019-0499-1

**Published:** 2019-03-29

**Authors:** Shuangshuang Yang, Haofeng Xu, Jide Sun, Shan Sun

**Affiliations:** 1 Department of Laboratory Medicine, the First Affiliated Hospital of Chongqing Medical University, No.1, Youyi Road, Yuzhong District, Chongqing, 400016 People’s Republic of China; 2 Laboratory Medicine, Chongqing Medical University, No.1, Yixueyuan Road, Yuzhong District, Chongqing, 400016 People’s Republic of China

**Keywords:** Bloodstream infections, ESKAPEEc, Antibiogram, Time trend, Pediatric, Adult

## Abstract

**Background:**

ESKAPEEc contribute to a majority of bloodstream infections (BSIs) and their antibiogram have changed overtime, while data concerning about these alterations are lacking in China. Added that a paucity of studies referred to ESKAPEEc in pediatric BSIs, our study aimed to demonstrate the longitudinal alterations of ESKAPEEc distribution and antibiogram in adult and pediatric BSIs in Southwest China.

**Methods:**

A multicenter retrospective surveillance study was launched from 2012 to 2017. Data of China Antimicrobial Resistance Surveillance System (CARSS) was analyzed by Whonet 5.6 and Graphpad Prism 6 Software. *Chi-square* test or *Fisher’s* exact test was used to examine and compare temporal changes.

**Results:**

A total of 32,259 strains was isolated, with 17.4% from pediatric BSIs. ESKAPEEc contributed to 58.67% (18,924/32,259) of BSIs, with 65.3% of adult BSIs and 27.2% of pediatric BSIs. *Escherichia coli* (*E. coli*) and *Klebsiella pneumoniae* (*K. pneumoniae*) were the two predominant species. Carbapenem resistance was prevalent in 0.76, 4.60, 9.47,13.66, 59.47% of *E. coli*, *K. pneumoniae*, *Enterobacter cloacae*, *Pseudomonas aeruginosa* (*P. aeruginosa*) and *Acinetobacter baumannii* (*A. baumannii*), respectively. The proportions of methicillin-resistant *Staphylococcus aureus* (MRSA) and vancomycin-resistant *Enterococcus faecium* (VREFM) were 28.91% and 2.20%, respectively. Between 2012-2014 and 2015–2017, *E. coli* and *K. pneumonia* showed significantly increased resistance rates to imipenem but decreased to ceftriaxone and ceftazidime, while *A. baumannii* exhibited reduced resistances to almost all the beta-lactams tested. The prevalence of antimicrobial resistance to most of agents against Gram-positive ESKAPEEc did not significantly varied during the same timeframe. In comparison with those from adult BSIs, *K. pneumoniae* from pediatric BSIs exhibited high resistance rates to all the beta-lactams tested, especially to carbapenems (12.79% vs 3.87%), while *A. baumannii* showed low resistance rates to all the agents.

**Conclusions:**

Ongoing burden of ESKAPEEc in BSIs and increasing trend of imipenem resistance in *E. coli* and *K. pneumoniae* call for continued surveillance. Carbapenems are still active against Gram-negative ESKAPEEc, except for *A. baumannii* and vancomycin or linezolid is still effective against Gram-positive ESKAPEEc. Carbapenem-resistant *K. pneumoniae* in children and carbapenem-resistant *A. baumannii* in adults necessitate effective antimicrobial strategies in consideration of age stratification.

## Background

Sepsis has been recognized as a global health priority by world health organization (WHO) in 2017. Better assessment of antimicrobial resistance (AMR) of pathogens causing bloodstream infections (BSIs) is one of the most significant resolutions to stem the tide of BSIs and thus alleviate the burden of sepsis [1–3]. ESKAPEEc, a cluster of notorious pathogens including *Enterococcus faecium*, *Staphylococcus aureus*, *Klebsiella pneumoniae*, *Acinetobacter baumannii*, *Pseudomonas aeruginosa*, *Enterobacter spp* and *Escherichia coli*, have been reported to contribute 50–70% of all causative pathogens to BSIs [4–6]. The striking emergence and transmission of extensive beta-lactamases (ESBLs) or carbapenemase genes among Gram-negative robs have vigorously driven the prominence of Gram-negative ESKAPEEc in BSIs. In addition, due to the implement of vaccination campaigns and comprehensive antimicrobial stewardship activities, a shift in the distribution and AMR patterns of ESKAPEEc was ongoing worldwide [7–11]. However, data concerning about these changes in BSIs were lacking in China.

Moreover, foregoing studies failed to discuss these alterations in pediatric populations. A longitudinal surveillance study in United States indicated that children witnessed an increased prevalence of carbapenem-resistant Enterobacteriaceae (CRE) in BSIs from 0.0% in 1999–2000 to 4.5% in 2011–2012 [12]. Recently, national surveillance data from China in 2013 had reported that pediatric patients witnessed highest prevalence of carbapenem resistance in *Klebsiella pneumoniae* (10.6%) [4]. However, a paucity of data has referred to the shifting of this high prevalence among pediatric BSIs over time in China.

To fill this gap, we launched a 6-year retrospective multicenter study from 2012 to 2017 to elucidate the longitudinal alterations of pathogen distributions and antibiogram of ESKAPEEc among pediatric and adult BSIs in Chongqing, Southwest China. Age distributions and AMR patterns were further compared between 2012–2014 and 2015–2017 two periods.

## Methods

### Study design and data enrollment criteria

This retrospective study was based on blood-culture proven BSIs and conducted in the first affiliated hospital of Chongqing Medical University, a branch of the China Antimicrobial Resistance Surveillance System (CARSS) in Southwest China, which included 54 microbiological laboratories from secondary or tertiary hospitals in Chongqing. Data of all the isolates were collected from the database of CARSS. Isolates were enrolled if specifically reported all the information including patients’ age, unique patient identification number and antibiotic susceptibilities with minimal inhibitory concentration (MIC) values. According to the Clinical Laboratory Standards Institute (CLSI) M39-A4, only the first isolate was evaluated in this present study, in consideration that inclusion of multiple isolates from an individual patient who has successive blood culture series, several episodes of different BSIs during hospitalization or poly-microbial BSIs may overestimate the risk of acquiring a resistant strain [13, 14].

### Bacteria identification and antimicrobial susceptibility

All participated laboratories followed standard procedures to fulfill blood culture, bacteria identification, and antimicrobial susceptibility testing on semi- or automated systems, according to the guidelines of CLSI. Antimicrobial susceptibilities of bacteria were interpreted by the recommendation of CLSI-M100 S27.

### Definitions

Children was defined if patients were no elder than 14 years old, while adult was defined if patients were elder than 14 years old [15].

ESKAPEEc was a cluster of pathogens including *Enterococcus faecium* (*E. faecium*), *Staphylococcus aureus* (*S. aureus*), *Klebsiella pneumoniae* (*K. pneumoniae*), *Acinetobacter baumannii* (*A. baumannii*), *Pseudomonas aeruginosa* (*P. aeruginosa*), *Enterobacter cloacae* (*E. cloacae*) and *Escherichia coli* (*E. coli*) [5].

Carbapenem resistance of *K. pneumoniae*, *E. cloacae* or *E. coli* strains was defined as resistance to at least one of carbapenems: ertapenem (MIC≥2), imipenem (MIC≥4) or meropenem (MIC≥4), while that of *A. baumannii* or *P. aeruginosa* strains was defined if resistance to either imipenem (MIC≥8) or meropenem (MIC≥8).

### Statistical analysis

Raw data was firstly processed by Whonet 5.6 software and then calculated on Graphpad prism 6 and SPSS v.21.0 (SPSS, Chicago, IL, USA) software. Temporal changes in age distributions and AMR were further determined by *Chi-square* test or *Fisher’s* exact test. Statistical significance was confirmed if a two-tailed *P* value was no more than 0.05.

## Results

### Alterations of ESKAPEEc distribution in BSIs

**Fig. 1:**
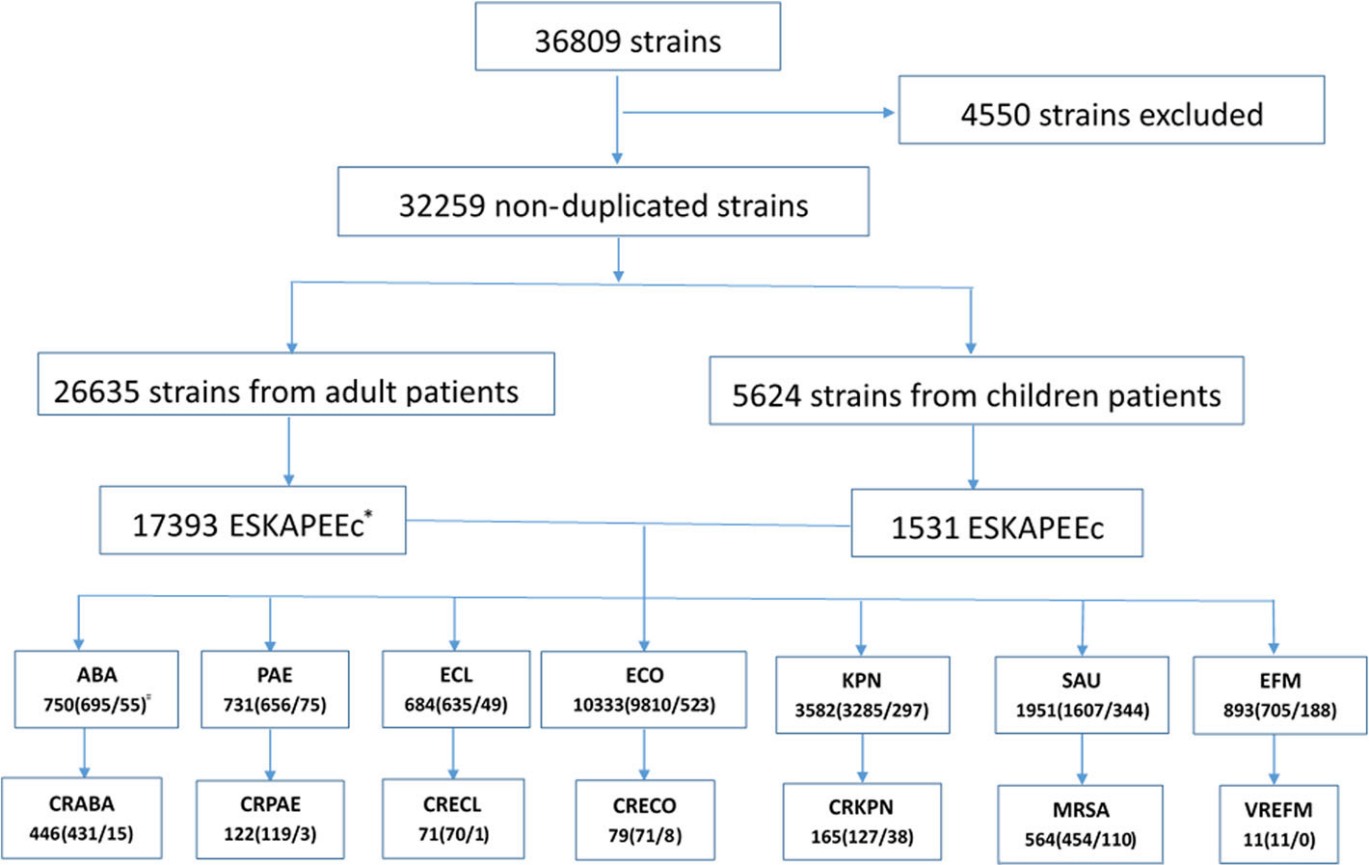
ESKAPEEc pathogens encountered in blood culture specimens in this present study. ESKAPEEc*: *Enterococcus faecium* (EFM), *Staphylococcus aureus* (SAU), *Klebsiella pneumoniae* (KPN), *Acinetobacter baumannii* (ABA), *Pseudomonas aeruginosa* (PAE), *Enterobacter cloacae* (ECL) and *Escherichia coli* (ECO); CRABA: carbapenem-resistant *Acinetobacter baumannii*; CRPAE: carbapenem-resistant *Pseudomonas aeruginosa*; CRECL: carbapenem-resistant *Enterobacter cloacae*; CRECO: carbapenem-resistant *Escherichia coli*; CRKPN: carbapenem-resistant *Klebsiella pneumoniae*; MRSA: methicillin-resistant *Staphylococcus aureus*; VREFM: Vancomycin-resistant *Enterococcus faecium*. N (A/B) ^**#**^: N is the total number of strains from BSIs. A is the number of strains isolated from adult BSIs, while B is that from pediatric BSIs

**Fig. 2:**
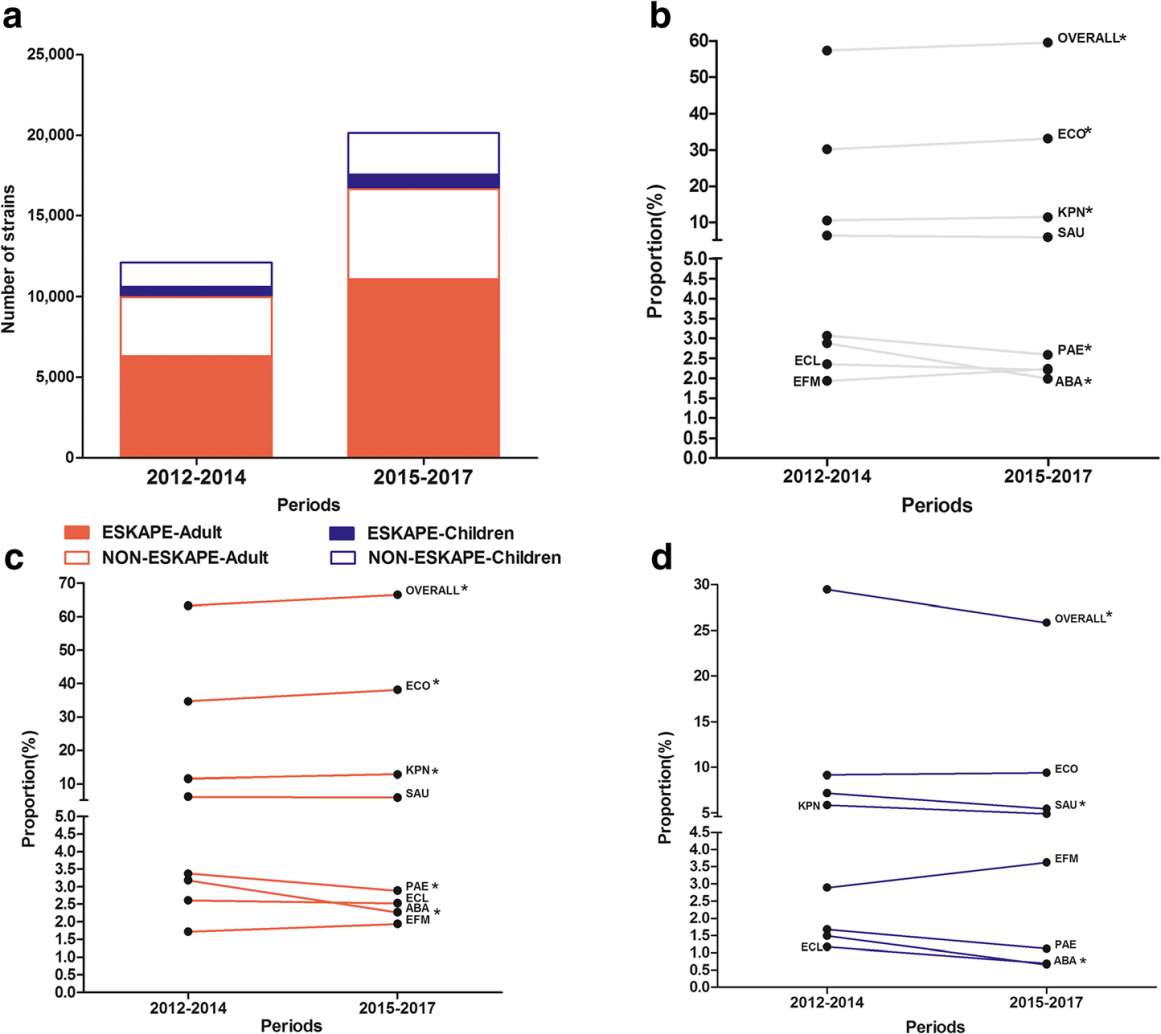
Time trend and age distribution of ESKAPEEc pathogens in bloodstream infections (BSIs) from 2012 to 2014 to 2015–2017. **a** General distribution of ESKAPEEc pathogens in BSIs; **b** Constitutions of ESKAPEEc pathogens in BSIs; **c** Constitutions of ESKAPEEc pathogens in adult BSIs; **d** Constitutions of ESKAPEEc pathogens of pediatric BSIs. Asterisks indicate statistical significance as *P* values were less than 0.05

Between 2012 and 2017, a total of 36,809 strains was isolated from blood culture specimens. Of them, 32,259 strains were included according to the enrollment criteria (Fig. 1). ESKAPEEc accounted for 58.67% (18,924/32,259) of Blood-borne pathogens. As shown in Table 1, *E. coli* (32.03%) was the most frequently isolated bacteria, followed by *K. pneumoniae* (11.10%), *Staphylococcus epidermidis* (10.23%) and *S. aureus* (6.05%). The total number of strains increased from 12,114 in 2012–2014 to 20,145 in 2015–2017 (Fig. 2a) and the corresponding proportion of ESKAPEEc significantly increased from 57.3% in 2012–2014 to 59.5% in 2015–2017 (*χ*^*2*^ = 15.74, *P* < .0001), largely due to the dramatical rise of *E. coli* (30.15% vs 33.16%) and *K. pneumoniae* (10.52% vs 11.46%) in isolation rate. During the same time frame, the percentages of *A. baumannii* (2.88% vs 1.99%) and *P. aeruginosa* (3.07% vs 2.59%) were decreased (Fig. 2b).

**Table 1:** Age distribution of TOP 10 pathogens isolated from blood culture specimens, 2012–2017

Pathogens	Overall			Adults			Children		
	NO of pathogens (*n* = 32259)	Percentage	Rank	NO of pathogens (*n* = 26635)	Percentage	Rank	NO of pathogens (*n* = 5624)	Percentage	Rank
Gram-negative									
*Escherichia coli*	10333	32.03%	1	9810	36.83%	1	523	9.30%	1
*Klebsiella pneumoniae*	3582	11.10%	2	3285	12.33%	2	297	5.28%	2
*Pseudomonas aeruginosa*	893	2.77%	7	818	3.07%	3	75	1.33%	3
*Enterobacter cloacae*	749	2.32%	9	700	2.63%	4	49	0.87%	5
*Acinetobacter baumannii*	750	2.32%	8	695	2.61%	5	55	0.98%	4
Gram-positive									
*Staphylococcus epidermidis*	3300	10.23%	3	1713	6.43%	1	1587	28.22%	1
*Staphylococcus aureus*	1951	6.05%	4	1607	6.03%	2	344	6.12%	4
*Staphylococcus hominis*	1924	5.96%	5	1316	4.94%	3	608	10.81%	2
*Staphylococcus haemolyticus*	1231	3.82%	6	803	3.01%	4	428	7.61%	3
*Enterococcus faecium*	687	2.13%	10	499	1.87%	5	188	3.34%	6
*Streptococcus pneumoniae*	486	1.51%	12	262	0.98%	13	224	3.98%	5

ESKAPEEc were attributable to 65.3% of all causative pathogens in adult BSIs and 27.2% in pediatric BSIs, and showed different shifting patterns in adult and pediatric BSIs from 2012 to 2014 to 2015–2017. Briefly, there was a significant increase in the prevalence of ESKAPEEc in adult BSIs (63.23% vs 66.54%, *P* < .0001, Fig. 2c), which may be primarily due to the dramatically increased proportions of *E. coli* and *K. pneumoniae*. In contrast, there was a decreasing trend in pediatric BSIs (29.46% vs 25.85%, *P* = .0035, Fig. 2d), which may be mainly ascribed to the sharp drop in the proportions of *A. baumannii* and *S. aureus* during the same periods.

### Antimicrobial resistance (AMR) patterns of ESKAPEEc by time

**Fig. 3:**
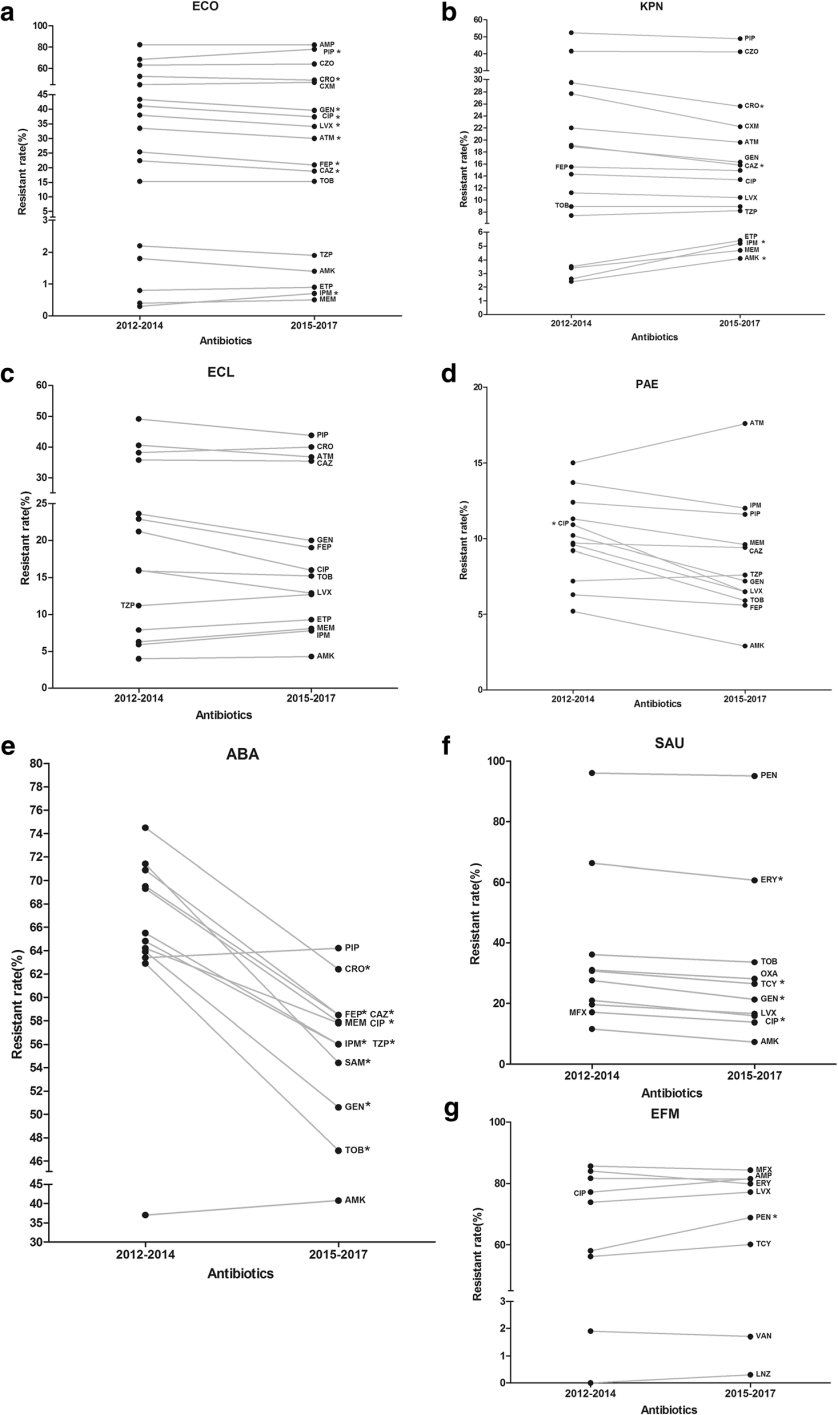
Alterations in antimicrobial resistance profiles of ESKAPEEc pathogens from 2012 to 2014 to 2015–2017. **a** ECO, *Escherichia coli;* **b** KPN, *Klebsiella pneumoniae*; **c** ECL, *Enterobacter cloacae*; **d** PAE, *Pseudomonas aeruginosa*; **e** ABA, *Acinetobacter baumannii*; **f** SAU, *Staphylococcus aureus*; **g** EFM, *Enterococcus faecium*. PEN: Penicillin G; AMP: Ampicillin; OXA: Oxacillin; PIP: Piperacillin; TZP: Piperacillin/Tazobactam; CZO: Cefazolin; CXM: cefuroxime; CAZ: Ceftazidime; CRO: Ceftriaxone; FEP: Cefepime; ATM: Aztreonam; ETP: Ertapenem; IMP: Imipenem; MEM: Meropenem; CIP: Ciprofloxacin; LEV: Levofloxacin; MXF: Moxifloxacin; AMK: Amikacin; GEN: Gentamicin; TOB: Tobramycin; ERY: Erythromycin; CLI: clindamycin; CHL: chloramphenicol; TCY: Tetracycline; LZD: Linezolid; VAN: Vancomycin. Asterisks indicate statistical significance as *P* values were less than 0.05

Since an increasing incidence of ESKAPEEc was observed over time, the changes in AMR profiles were further investigated. Among *E. coli*, the resistant rates to ceftriaxone, ceftazidime, cefepime, aztreonam, gentamicin, ciprofloxacin and levofloxacin were markedly decreased, while those to piperacillin and imipenem were dramatically increased during two studied periods from 2012–2014 to 2015–2017 (Fig. 3a). Likewise, the resistant rates of *K. pneumoniae* to ceftriaxone and ceftazidime were markedly decreased, while the resistant rates to imipenem and amikacin were significantly increased during the same periods (Fig. 3b). *E. cloacae* showed no significant alterations in the resistance rates to all the studied antibiotics and similar tendency was also found in *P. aeruginosa*, except for reduced resistance to ciprofloxacin (Fig. 3c & d). Of note, *A. baumannii* showed a sharp reduction in the resistance to a majority of antibiotics tested, especially to imipenem (From 65.5 to 56.0%), whereas its resistance to meropenem, amikacin and piperacillin remained stable (Fig. 3e).

As for Gram-positive ESKAPEEc, *S. aureus* exhibited reduced resistance to gentamicin, ciprofloxacin, erythromycin and tetracycline, while its resistance to oxacillin remained stable, near to 30% (Fig. 3f). For *E. faecium*, resistance rates to all the agents tested did not significantly vary overtime, except for increased resistance to penicillin G from 58.0 to 68.9% (Fig. 3g).

### Antimicrobial resistance (AMR) patterns of gram-negative ESKAPEEc by age

**Fig. 4:**
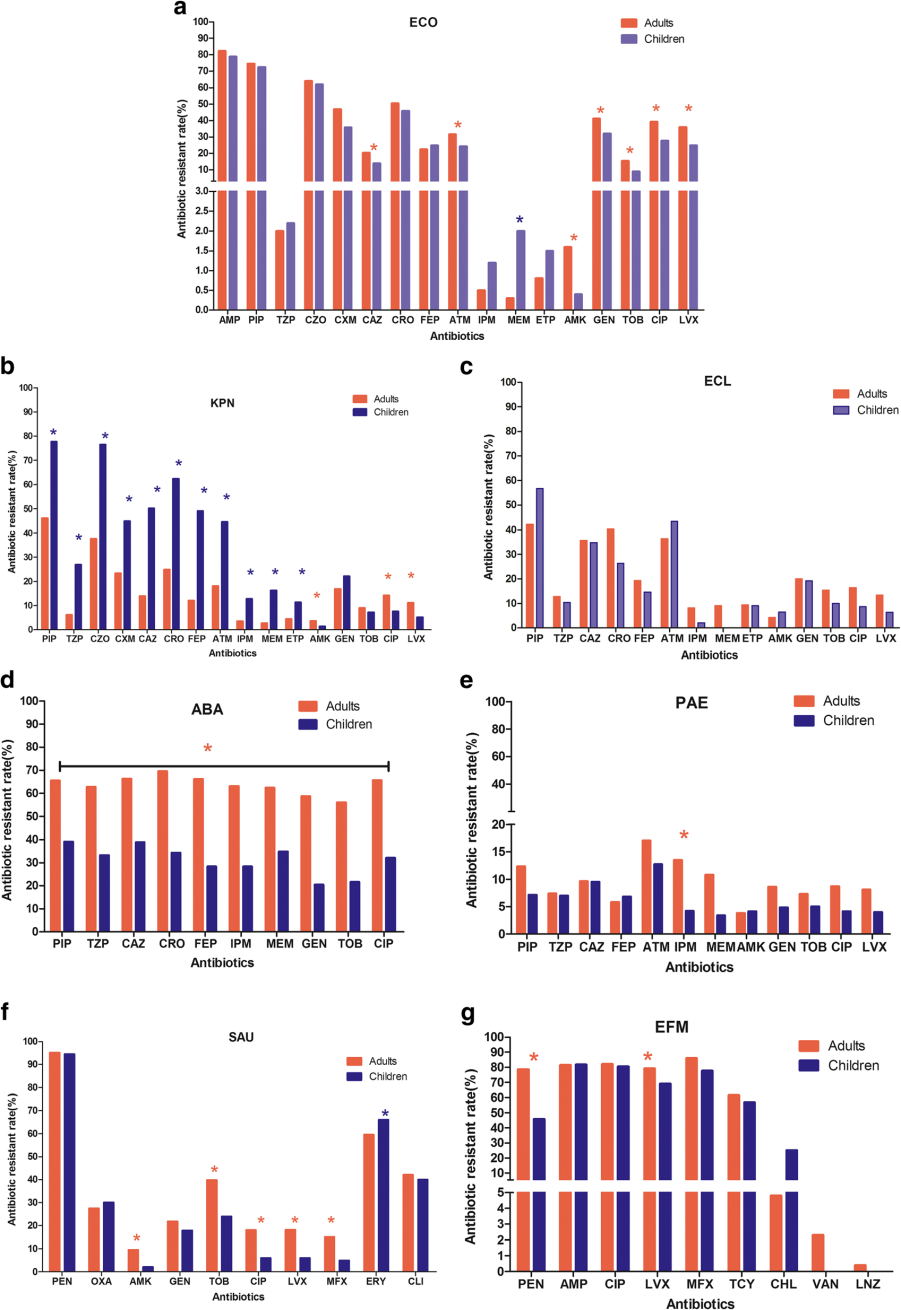
Antimicrobial resistance profiles of ESKAPEEc pathogens in adult and pediatric BSIs. **a** ECO, *Escherichia coli*; **b** KPN, *Klebsiella pneumoniae*; **c** ECL, *Enterobacter cloacae*; **d** ABA, *Acinetobacter baumannii*; **e** PAE, *Pseudomonas aeruginosa*; **f** SAU, *Staphylococcus aureus*; **g** EFM, *Enterococcus faecium*. PEN: Penicillin G; AMP: Ampicillin; OXA: Oxacillin; PIP: Piperacillin; TZP: Piperacillin/Tazobactam; CZO: Cefazolin; CXM: cefuroxime; CAZ: Ceftazidime; CRO: Ceftriaxone; FEP: Cefepime; ATM: Aztreonam; ETP: Ertapenem; IMP: Imipenem; MEM: Meropenem; CIP: Ciprofloxacin; LEV: Levofloxacin; MXF: Moxifloxacin; AMK: Amikacin; GEN: Gentamicin; TOB: Tobramycin; ERY: Erythromycin; CLI: clindamycin; CHL: chloramphenicol; TCY: Tetracycline; LZD: Linezolid; VAN: Vancomycin. Asterisks indicate statistical significance as *P* values were less than 0.05

Since distributions of ESKAPEEc differed between adult and pediatric BSIs, we hypothesized different antibiogram of ESKAPEEc in adult and pediatric BSIs. Concerning *E. coli*, carbapenem resistance was more prevalent in pediatric BSIs than in adult BSIs. The prevalence of meropenem resistance in pediatric BSIs (2.0%) was notably higher than that in adult BSIs (0.3%) (*P* < .0001, Fig. 4a). However, resistance rates to ceftazidime and aztreonam in pediatric BSIs were lower than those in adult BSIs. No significant difference in the prevalence of ceftriaxone and cefepime resistance was observed between these two groups, ranging from 45.8 to 50.5% and 24.9 to 22.4%, respectively.

In contrast to *E. coli*, *K. pneumoniae* showed different AMR profiles between these two groups. Isolates from pediatric BSIs exhibited markedly high resistance rates to all beta-lactam agents tested, compared to those from adult BSIs (Fig. 4b). In brief, 49.0 to 62.4% of isolates from pediatric BSIs, but 12.1 to 24.9% from adult BSIs were resistant to ceftriaxone, ceftazidime and cefepime. Noticeably, carbapenem resistance prevalence was 12.79% among pediatric isolates, which was higher than 3.87% among adult BSIs (*P*<. 0001).

Unlike *E. coli* and *K. pneumoniae*, no significant difference in AMR profiles of *E. cloacae* was found between adult and pediatric BSIs (Fig. 4c). Resistance rates to piperacillin/tazobartan, cefepime, amikacin, ciprofloxacin, levofloxacin and tobramycin were less than 20%. However, it was noteworthy that carbapenem resistance among *E. cloacae* was 9.47%.

As to non-fermenters, *A. baumannii* isolated from adult BSIs showed significantly higher resistance ratios to all the agents tested than those from pediatric BSIs (all *P* < .05, Fig. 4d). The prevalence of resistance to all these agents was approximately 60% in adult BSIs, especially to carbapenems (62.01%), while that against *P. aeruginosa* was less than 20%. Although *P. aeruginosa* isolated from adult BSIs showed higher resistance to almost all these antibiotics, only the prevalence of imipenem resistance was statistically higher than those from pediatric BSIs (13.5% vs 4.2%, *P* < .05, Fig. 4e).

### Antimicrobial resistance (AMR) patterns of gram-positive ESKAPEEc by age

In terms of Gram-positive ESKAPEEc, the prevalence of MRSA was similar among adult and pediatric BSIs (28.25% vs 31.98%). *S. aureus* isolated from adult BSIs illustrated significantly higher resistance rate to moxifloxacin (15.1% vs 5.0%), while isolates from children BSIs showed higher resistance rate to erythromycin (59.6% vs 66.1%, Fig. 4f). None of *S. aureus* isolated from either group was resistant to vancomycin or linezolid. As to *E. faecium*, resistance rates to ampicillin, ciprofloxacin, levofloxacin and moxifloxacin ranged from 69.0 to 85.9%. Eleven isolates were resistant to vancomycin and all of them were isolated from adult, with a ratio of 2.20%. Penicillin resistance among pediatric BSIs was 45.7%, which was significantly lower than 78.5% from adult BSIs (*P* < .01). Similar trend was also observed in levofloxacin resistance (69.0% vs 79.2%, *P* < .05, Fig. 4g).

## Discussions

To our knowledge, this study presented the current available evidence of the time shifting of ESKAPEEc distributions and AMR patterns in children and adult BSIs from China. Our findings verified the increasing prevalence of ESKAPEEc in BSIs and their distinct AMR patterns in adult and pediatric BSIs.

ESKAPEEc contributed to 58.67% of BSIs, which was consistent with previous studies [5, 16]. Our observation of an overall increasing trend of ESKAPEEc in BSIs was largely due to the striking upward trend of *E. coli* and *K. pneumoniae* in adult BSIs. Similar results were also concluded by a 9-year analysis of BSIs in Rome [5]. However, a recent long-term surveillance study at an urban hospital in Malawi showed a significant declination in the incidence of Enterobacteriaceae in BSIs from 1998 to 2016 [7]. This discrepancy was partly due to the heterogeneity of study design, since this present study was conducted in laboratories of more than 40 hospitals and had greater geographical representation.

Interestingly, in accordance with an 11-year retrospective study in US [17], ESKAPEEc accounted for 27.2% of all causative pathogens in pediatric BSIs, but we firstly observed a sharp drop in the prevalence of ESKAPEEc in pediatric BSIs in China, mainly driven by the slight reduction of *A. baumannii* and *S. aureus*. This drop may suggest that other successful species are prevalent in pediatric BSIs, but we noticed that CoNS was predominant in this present study and its predominance may override the prevalence of ESKAPEEc. Added that, data from Typhoid Surveillance in Africa Program of Sub-Saharan Africa had verified that children had significantly high odds of having a contaminated blood culture compared with adults [15]. Therefore, we deduced that blood contamination should be blamed for our high incidence of CoNS and decreased incidence of ESKAPEEc in pediatric BSIs, even though that previous studies in the US and Chongqing also reported CoNS was the most frequently isolated from pediatric BSIs [17, 18].

Unexpectedly, decreased resistance rates to ceftriaxone and ceftazidime but increased resistant rates to imipenem were observed among *E. coli* and *K. pneumoniae*. Similar trends were also observed by Lei Tian et al. [19]. The possible explanation of this alteration was that preferential antibiotic adoption of imipenem led to antibiotic selective pressure. Since ESBLs are notorious in nosocomial infection, most of empirical antibiotic therapies of BSIs are initiated with imipenem other than the third- or four- generation cephalosporins. Then, high consumption leads to high resistance. Recent studies in China and abroad have demonstrated that increased usage of carbapenems accelerates the production of carbapenemase, and then high proportion of CRKP [20–22].

Of note, our study alarmed high prevalence of CRKP (12.79%) among pediatric BSIs, which was higher than data reported by CHINET [4]. However, a comparative study of neonatal BSIs in Chongqing revealed that all of 49 strains were susceptible to imipenem [18]. Our high prevalence may due to the heterogeneity of resistant element among strains and the dissemination of conservative mobile elements. Nationwide surveillance of CRE in China had confirmed carbapenemase production was the primary mechanism of carbapenem resistance in CRE. The mobile element of *ISKpn27-blaKPC-2-ISKpn2* played an important role in CRKP transmission [23]. However, in pediatric patients, several studies have reported the predominance or outbreak of *bla*_NDM-1_ among CRKP in Beijing, Shanghai and Shandong [24–26]. Our previous studies also demonstrated a high prevalence of *bla*_NDM-1_ among CRKP in Chongqing [27, 28]. Therefore, more researches are ongoing to uncover the underlying molecular mechanisms of the high prevalence of CRKP in children BSIs and cautious adoption of carbapenems to fight against pediatric CRKP BSI is advocated.

The prevalence of carbapenem resistance among *E. coli* was 0.76%, which was higher than results from an epicenter of CRE in the US with a ratio of 0.1% [29]. Moreover, it was noteworthy that eight CRECO strains were isolated from pediatric BSIs, suggesting the emergence of carbapenem resistance phenotypes among pediatric inpatients in Chongqing. Despite this, carbapenems or piperacillin/tazobactam are still active in our settings; less than 3% strains were resistant to them.

Accompanied with declined proportion of *A. baumannii* in BSIs, reduced resistance rates to most of antibiotics were also observed, especially to carbapenems (From 63.61 to 55.86%). This rate of carbapenem resistance was lower than our previous study suggesting 71.19% of CRAB in BSIs [30], but similar decreasing trend was reported in Dhaka [9]. This reduction may be owing to the implementation of CRASS strategies to fight against carbapenem-resistant robs, such as resistance surveillance, colonization clearance and environmental monitoring. However, it was noticed that in spite of this decreasing trend, resistance rates of *A. baumannii* to all these antibiotics were 60% in adult BSIs and 25% in pediatric BSIs, respectively, hence, effective treatments to fight against *A. baumannii* BSIs are still in urgent need. Recently, Tigecycline Evaluation and Surveillance Trial (TEST) concerning global blood-borne pathogens reported 70.8% of *A. baumannii* were susceptible to minocycline and MIC_90_ of tigecycline against it was 2 μg/mL, suggesting the potential efficiencies of these two antibiotics to overcome *A. baumannii* BSIs [13].

Moreover, TEST further reported high prevalence of MRSA and VREFM worldwide, with the proportions of 33.0 and 27.6%, respectively [13], while our study observed relatively low prevalence, with 28.91 and 2.2%, respectively. In contradistinction to the sharp reduction of MRSA in US [10], our finding suggested the proportion of MRSA remains constant. Despite penicillin resistance was dramatically increased and ampicillin resistance was consistently high among *E. faecium*, vancomycin or linezolid is still efficient to fight against Gram-positive ESKAPEEc in our settings.

Several limitations should be considered in our study. Firstly, although this study included almost all the microbiology laboratories in the secondary or tertiary hospitals in Chongqing, Southwest China, it excluded some laboratories which failed to perform antimicrobial susceptibility testing. Careful interpretation of our findings was advocated in different hospital settings. Secondly, this multicenter study was focused on laboratory based, blood-culture proven BSIs attributable to ESKAPEEc, it, therefore, was not designed to analyze blood culture contamination and to discuss hospital-onset or community-onset without sufficient clinical patient data. Thirdly, our study excluded the isolates with antimicrobial susceptibility results reported by Kirby-Bauer, which might lead to an overestimation of the incidences of antimicrobial resistance.

## Conclusions

Distributions and AMR patterns of ESKAPEEc had changed overtime and differed between adult and pediatric BSIs. We noted an ongoing burden of ESKAPEEc in BSIs, largely driven by an upward trend in adult BSIs. The two predominant species, *E. coli* and *K. pneumoniae* illustrated decreased resistances to ceftriaxone and ceftazidime but increased resistances to imipenem in the past 6 years. Carbapenems may be still efficient to fight against *E. coli-* and *K. pneumoniae-* BSIs, but it is noteworthy that high prevalence of CRKP in pediatric BSIs may wreck their efficiencies. Moreover, a decreasing tendency should not relax our vigilance of CRAB in consideration of their high resistance to all these first-line antibiotics and minocycline or tigecycline may be helpful to treat *A. baumannii* BSIs. AMR profiles of Gram-positive ESKAPEEc remained stable and vancomycin or linezolid remains active against them. Further stratified by age, most of ESKAPEEc from pediatric BSIs showed lower resistance rates to the first-line antimicrobials, except for *E. coli* and *K. pneumoniae*, which exhibited higher resistance rates to meropenem than isolates from adults, suggesting that AMR patterns of ESKAPEEc are associated with age. Taking into account age during the continuous surveillance and infection control of ESKAPEEc in BSIs was necessitated in Chongqing, Southwest China.

## Abbreviations

AMR: Antimicrobial resistance
BSIs: Bloodstream infections
CARSS: China Antimicrobial Resistance Surveillance System
CLSI: Clinical and Laboratory Standards Institute
CoNS: Coagulase-negative staphylococcus
CRABA: Carbapenem-resistant *Acinetobacter baumannii*
CRE: Carbapenem-resistant Enterobacteriaceae
CRECL: Carbapenem-resistant *Enterobacter cloacae*
CRECO: Carbapenem-resistant *Escherichia coli*
CRKP: Carbapenem-resistant *Klebsiella pneumoniae*
CRPAE: Carbapenem-resistant *Pseudomonas aeruginosa*
ESKAPEEc: *Enterococcus faecium*, *Staphylococcus aureus*, *Klebsiella pneumoniae*, *Acinetobacter baumannii*, *Pseudomonas aeruginosa*, *Enterobacter spp (*only *Enterobacter cloacae* included in this present study) and *Escherichia coli*
MIC: Minimum inhibitory concentration
MRSA: Methicillin-resistant *Staphylococcus aureus*
TEST: Tigecycline Evaluation and Surveillance Trial
VREFM: Vancomycin-resistant *Enterococcus faecium*
WHO: World health organization

## Antimicrobials

PEN: Penicillin G
AMP: Ampicillin
OXA: Oxacillin
PIP: Piperacillin
TZP: Piperacillin/Tazobactam
CZO: Cefazolin
CXM: Cefuroxime
CAZ: Ceftazidime
CRO: Ceftriaxone
FEP: Cefepime
ATM: Aztreonam
ETP: Ertapenem
IMP: Imipenem
MEM: Meropenem
CIP: Ciprofloxacin
LEV: Levofloxacin
MXF: Moxifloxacin
AMK: Amikacin
GEN: Gentamicin
TOB: Tobramycin
ERY: Erythromycin
CLI: Clindamycin
CHL: Chloramphenicol
TCY: Tetracycline
LZD: Linezolid
VAN: Vancomycin
